# Scapholunate Dissociation due to an Atypical Biomechanical Mechanism in Arm Wrestling: A Case Report

**DOI:** 10.1155/cro/3823464

**Published:** 2025-10-30

**Authors:** Fumikazu Niitsu, Manabu Tanaka, Itsuo Joko, Mio Uchida, Kazuo Kasuga, Shigeharu Uchiyama

**Affiliations:** Department of Orthopaedic Surgery, Okaya City Hospital, Okaya, Nagano, Japan

**Keywords:** arm wrestling, capitohamate bone–ligament–bone graft, scapholunate dissociation

## Abstract

**Background:**

Scapholunate ligament injuries are not uncommon in hand surgery, but they are not described in the sport of arm wrestling.

**Case Presentation:**

The patient was a 28-year-old man who experienced wrist pain when he was about to lose an arm wrestling match. Since then, he has had persistent clicking in his right wrist, which was found to be due to scapholunate dissociation. The reason for scapholunate dissociation rather than a humeral shaft fracture in our patient may be a reduction in torque on the humerus due to forced elbow extension, which could be seen in a losing player at the end of the match. This position could concentrate the reaction force on the wrist rather than on the humerus.

**Conclusions:**

Arm wrestling should be recognized as a potential cause of scapholunate dissociation due to its atypical injury pattern, necessitating early diagnosis. Furthermore, the unique biomechanics of arm wrestling warrant attention in orthopedic and surgical literature.

## 1. Introduction

Although arm wrestling is a popular sport, it can cause injuries to the upper extremity. Among these, the spiral fracture of the humeral shaft is well known, mainly due to the torsional force on the humerus [1, 2]. On the other hand, injuries to other sites, particularly the hand and wrist, are rare [3, 4]. We report a rare case of scapholunate dissociation caused by arm wrestling.

## 2. Case Report

**Figure 1:**
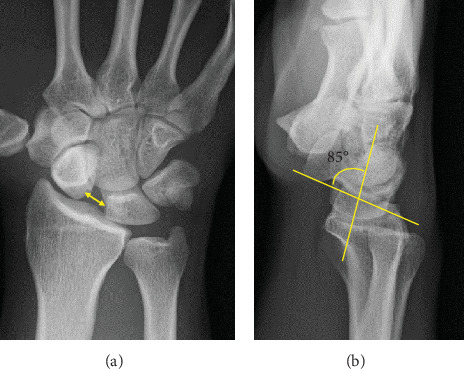
Preoperative plain radiograph of the right wrist. (a) Posteroanterior view demonstrates significant dissociation of the scapholunate interval (arrow) of 5.8 mm. (b) Lateral view demonstrates increased scapholunate angle of 85°.

**Figure 2:**
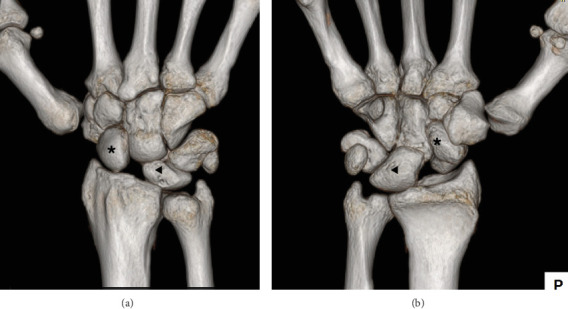
Preoperative 3DCT of the right wrist. (a) Posteroanterior view and (b) anteroposterior view demonstrate scapholunate dissociation with volarly flexed scaphoid (⁣^∗^) and dorsally extended lunate (◀︎).

**Figure 3:**
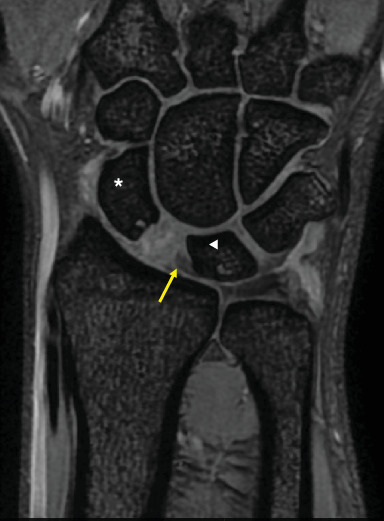
Preoperative MRI (T2⁣^∗^ 3D MERGE) of the right wrist. Coronal section demonstrates scapholunate dissociation with the attachment of the scapholunate ligament on the lunate side (arrow). ⁣^∗^: scaphoid, ◀︎: lunate.

A 28-year-old man developed pain in his right wrist while arm wrestling. He was not a competitive arm wrestler. He tried to force his opponent's hand down to the tabletop, but his opponent's strength overcame his, and he eventually lost the match. He felt a sudden pain in his right wrist as his opponent pushed his right hand down. He remembered that at the start of the match, his elbow was in 90° of flexion, forearm rotation was 0°, and the wrist was in a slightly flexed position. When he lost the match, the elbow was forcibly extended, and the dorsal side of the hand was grounded to the table. The pain in his right wrist improved over the next few months, but he still had a clicking sensation in his wrist. He was referred to our department 2 years after the injury because of persistent wrist crepitus. On initial presentation, the ROM of the right wrist was 70° in extension and 80° in flexion without restriction, and the Watson test was positive. There was no evidence of generalized joint laxity. A plain posteroanterior radiograph of the right wrist revealed significant dissociation of the scapholunate interval of 5.8 mm and a positive Terry–Thomas sign (Figure 1). Scapholunate dissociation was also identified using 3DCT and MRI scans, with the latter confirming the attachment of the scapholunate ligament on the lunate side (Figures 2 and 3). In lateral view, the scaphoid was flexed volarly and the lunate was dorsiflexed, and the scapholunate angle was increased to 85°. The lunate fossa inclination (angle between the sclerotic line of the lunate fossa of the radius and a line perpendicular to the long axis of the distal ulna) and radial inclination were within the normal range, and the diagnosis was chronic scapholunate dissociation. As it had been 2 years since the injury, it was considered difficult to suture the degenerated scapholunate ligament, and we planned to reconstruct the scapholunate ligament using a capitohamate bone–ligament–bone graft [5–7]. The patient underwent surgery under general anesthesia. Arthroscopic examination showed that the scapholunate ligament was torn circumferentially on the scaphoid side but remained on the lunate side. The cartilage at the distal end of the radius and carpi was normal. A 5-cm longitudinal incision was made on the dorsal aspect of the wrist to expose the dorsal carpal bones. The scapholunate ligament was considered viable, and four 2-0 absorbable sutures were placed from the volar to the dorsal side of the scaphoid, and the capitohamate ligament with bone was harvested and grafted over the scapholunate interval using two miniscrews.

Immediate postoperative radiographs showed normal carpal alignment with correction of volar flexion of the scaphoid and dorsiflexion of the lunate.

The postoperative course was uneventful. Range of motion exercises were started 8 weeks postoperatively. One year after surgery, the patient reported no pain or crepitus in the affected wrist. The ROM of the right wrist was limited to 60° of extension and 60° of flexion, but his grip strength had recovered from 36 to 54 kg, and the Watson test was negative.

**Figure 4:**
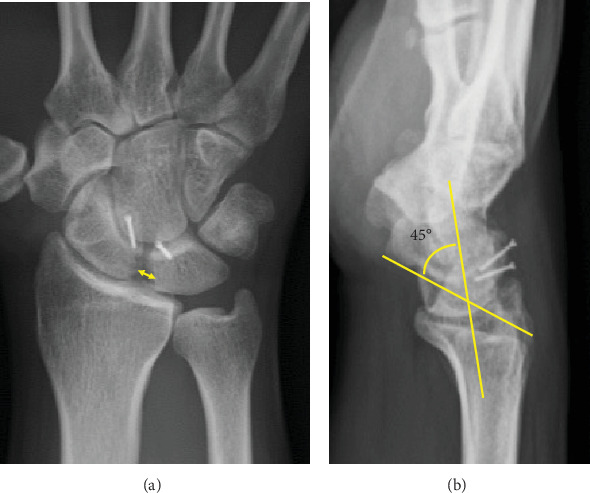
Plain radiograph of the right wrist at postoperative 1 year. (a) Posteroanterior view demonstrates normal scapholunate interval (arrow) of 2.5 mm. (b) Lateral view also demonstrates normal scapholunate angle of 45°. Two miniscrews are used to fix the capitohamate ligament with bone graft over the dorsal site of the scapholunate.

Radiographs of the right wrist showed improvement in the scapholunate interval from 5.8 to 2.5 mm and the scapholunate angle from 85° to 45° (Figure 4).

## 3. Discussion

Although a well-known serious injury in arm wrestling is the distal humeral spiral fracture, injuries can occur at sites other than the humeral shaft fracture, including avulsion of the long head of the biceps tendon from the glenoid [8], rupture of the subscapularis tendon [9], shoulder dislocation [10], scapular neck fracture [11], radial shaft fracture [12], radial neck fracture [13], rupture of the medial collateral ligament of the elbow [14], avulsion fracture of the medial humeral epicondyle [15], anterior dislocation of the elbow [16], fracture of the olecranon [17], subluxation of the extensor carpi ulnaris tendon [3], or rupture of the ulnar collateral ligament of the MP joint of the thumb [4]. These differences in injury sites and structures may be due to the position of the wrist, elbow, or shoulder and/or the strength of muscle contraction. Injuries around the wrist are very rare, and only subluxation of the extensor carpi ulnaris tendon has been reported. Although details of the mechanism of injury were not described, the authors speculated that the injury occurred due to hypersupination of the forearm [3].

**Figure 5:**
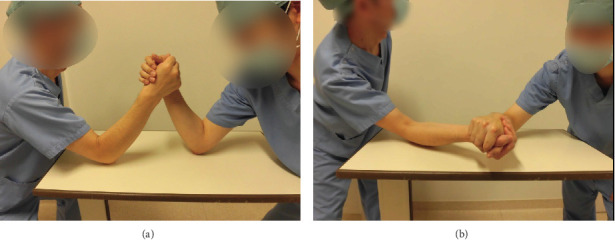
(a) Illustration of an arm wrestling match just before the contest. (b) The elbow extension and wrist extension are seen in the losing player at the end of the match.

According to Moloney et al. [2], the winning athlete rotates the shoulder internally by concentric contraction of the muscles around the shoulder. The elbow joint remains flexed and fixed, and the muscles undergo isometric contraction. At the end of the play, the wrist is flexed, and the pronators and flexors contract concentrically. The losing player, on the other hand, experiences an eccentric contraction of the internal rotators around the shoulder and finally an extension of the elbow and wrist joints (Figure 5). In other words, there are eccentric contractions of the elbow flexors, wrist flexors, and pronators.

Scapholunate ligament rupture is usually thought to occur when the hand is placed in wrist extension, ulnar deviation, and supination against the forearm and is torn as a result of the reaction force from the ground when the patient falls [18, 19]. This mechanism of action is thought to have been activated when the patient exerted force during arm wrestling. Our patient lost the match when he experienced pain in his right wrist, not his upper arm. Presumably, the elbow extension seen in the losing player at the end of the match may reduce the torque on the humerus and concentrate the reaction force to extension on the wrist rather than the humerus. This may be the reason why our patient experienced a scapholunate dissociation rather than a humeral fracture.

As our patient did not have any risk factors for scapholunate dissociation, such as low lunate inclination, low radial inclination of the distal radius [20], or generalized joint laxity [21], this injury occurred solely as a result of the force generated during arm wrestling, without any predisposing factors.

We used a capitohamate bone–ligament–bone graft for treatment to reconstruct the scapholunate dissociation [5, 6]. This resulted in satisfactory wrist function 1 year postoperatively. However, it is unclear whether it is the sutured scapholunate ligament, the capitohamate bone–ligament–bone graft, or both that are functioning. We have reported the operative technique and results for this patient elsewhere [7].

In conclusion, arm wrestling should be recognized as a potential cause of scapholunate dissociation due to its atypical injury pattern, necessitating early diagnosis. Furthermore, the unique biomechanics of arm wrestling warrant attention in orthopedic and surgical literature.

## Data Availability

The data that support the findings of this study are available from the corresponding author upon reasonable request.
